# Screening and identification of key biomarkers of depression using bioinformatics

**DOI:** 10.1038/s41598-023-31413-1

**Published:** 2023-03-13

**Authors:** Xinru Kong, Chuang Wang, Qiaolan Wu, Ziyue Wang, Yu Han, Jing Teng, Xianghua Qi

**Affiliations:** 1 Shandong University of Traditional Chinese Medicine, Jinan, 250355 Shandong China; 2 Affiliated Hospital of Shandong University of Traditional Chinese Medicine, Jinan, 250000 Shandong China

**Keywords:** Genetics, Molecular biology, Medical research, Molecular medicine, Pathogenesis

## Abstract

We aimed to identify the molecular biomarkers of MDD disease progression to uncover potential mechanisms of major depressive disorder (MDD). In this study, three microarray data sets, GSE44593, GSE12654, and GSE54563, were cited from the Gene Expression Omnibus database for performance evaluation. To perform molecular functional enrichment analyses, differentially expressed genes (DEGs) were identified, and a protein–protein interaction network was configured using the Search Tool for the Retrieval of Interacting Genes/Proteins and Cytoscape. To assess multi-purpose functions and pathways, such as signal transduction, plasma membrane, protein binding, and cancer pathways, a total of 220 DEGs, including 143 upregulated and 77 downregulated genes, were selected. Additionally, six central genes were observed, including electron transport system variant transcription factor 6, FMS-related receptor tyrosine kinase 3, carnosine synthetase 1, solute carrier family 22 member 13, prostaglandin endoperoxide synthetase 2, and protein serine kinase H1, which had a significant impact on cell proliferation, extracellular exosome, protein binding, and hypoxia-inducible factor 1 signaling pathway. This study enhances our understanding of the molecular mechanism of the occurrence and progression of MDD and provides candidate targets for its diagnosis and treatment.

## Introduction

Depression is a health-limited condition affecting around 280 million people worldwide^1^. Patients with depression experience disease symptoms for longer than two weeks, in contrast to emotional fluctuations and transitional emotional reactions to everyday life activities^2^, including sadness, irritability, weakness, loss of pleasure or interest in activities, inattention, excessive guilt or self-deprecation, despair of the future, death or suicide ideation, sleep disorder, appetite or weight change, and feeling particularly tired or lack of energy. According to a report^3^, over 700,000 suicides occur each year owing to depression-induced suicide, which is the fourth most common cause of death among people aged 15 to 29^4^.

Owing to the involvement of numerous biological, psychological, and social factors in the pathogenesis of depression, its etiology remains elusive. However, previous research indicates that genes are involved in depression. Major depressive disorder has been considered to exhibit a series of symptoms characterized by abnormal genetic segments on chromosomes in patients with persistent spontaneous depression^5^.

Microarray technology and bioinformatics analysis have been widely applied in genetic screening tests over the past few decades to help us identify differentially expressed genes (DEGs) and functional pathways associated with the diagnosis of depression^6^. However, it is challenging to obtain accurate results owing to the rate of false positives in an independent analysis by micro-networks. Therefore, in this study, three sets of mRNA microarray datasets were downloaded and analyzed from the Expression Omnibus (GEO) to obtain the DEG between postmortem brain tissues with and without depression.

## Materials and methods

**Figure 1:**
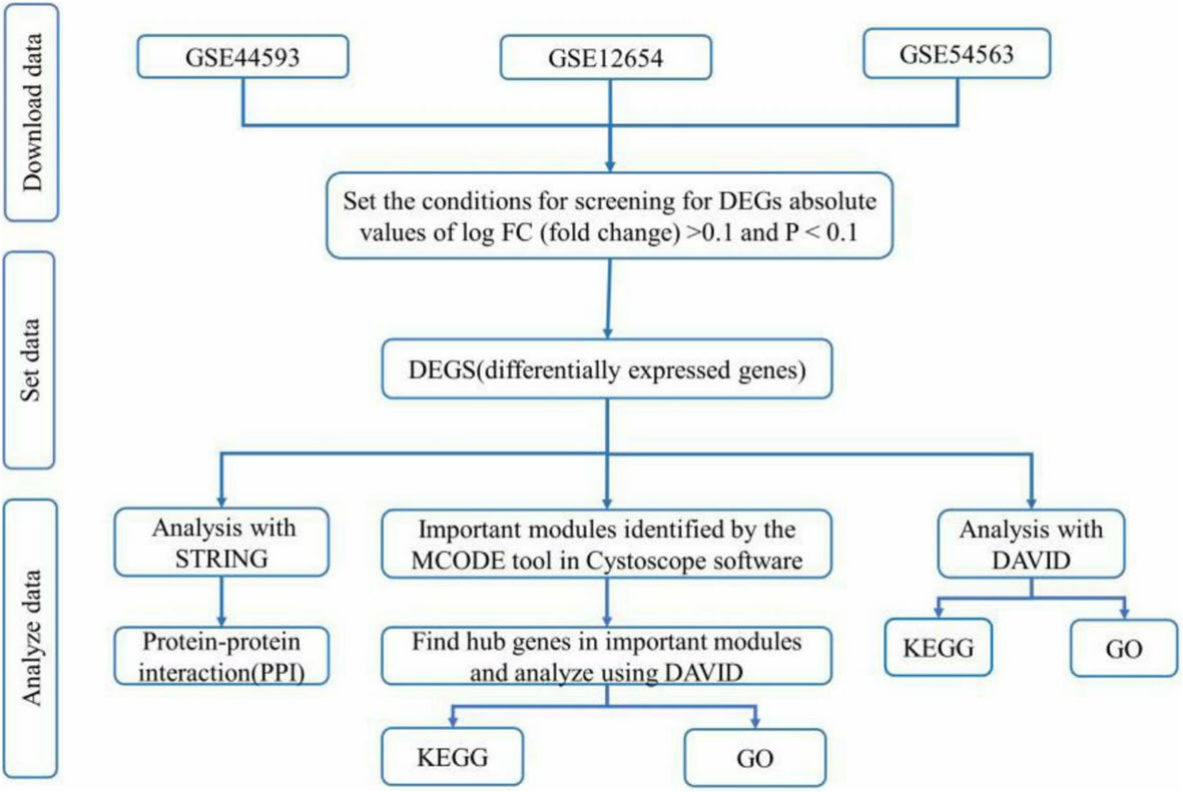
The flow chart analysis of the method.

To identify key biomarkers of depression, this study primarily used data from public functional genomic databases and bioinformatic technology. The flow chart analysis of the method is depicted in Fig. 1.

### Microarray data

GEO (http://www.ncbi.nlm.nih.gov/geo)^7^ is a public functional genomics database containing high-throughput gene expression data, chips, and microarrays. We observed three suitable brain specimens of patients with depression in the GEO database (there are relatively more blood samples of patients with depression; however, we believe that brain specimens are more in line with the selection of specimens for depression). Therefore, we selected GSE44593, GSE12654, and GSE54563. GEO is a gene expression database created and maintained by the National Center for Biotechnology Information (NCBI). It was founded in 2000 and contains high-throughput gene expression data submitted by research institutions worldwide. The published articles and the gene expression detection data involved in them can be obtained in this database. Three gene expression datasets, GSE44593^8^, GSE12654^9^, and GSE54563^10^, were downloaded from the GEO (Affymetrix GPL570 platform, Affymetrix Human Genome U133 Plus 2.0 Array). The probe is translated into the appropriate genetic symbol based on the platform annotation data. The GSE44593 (GPL570 (HG-saU133_Plus_2) Affymetrix Human Genome U133 Plus 2.0 Array) dataset contains 14 samples of cortical edge brain tissue and 14 samples from healthy people. GSE12654 (GPL8300 (HG-U95Av2) Affymetrix Human Genome U95 Version 2 Array) dataset contains 11 human prefrontal cortex samples and 11 human prefrontal cortex samples. The GSE54563 (GPL6947 Illumina HumanHT-12 V3.0 expression bead chip) database contains 25 human brain anterior cingulate samples from patients with major depressive disorder (MDD) and 25 of those from healthy people.

### Identification of DEGs

We used GEO2R (http://www.ncbi.nlm.nih.gov/geo/geo2r) to screen DEGs in people with MDD and without MDD. GEO2R is a data analysis tool that comes with the GEO database and can perform statistical analysis on gene expression profiles visually. GEO2R is an interactive network tool that compares two or more GEO series datasets to identify DEGs under experimental conditions^11^. The adjusted *P*-values (adj. *P*) and Benjamini and Hochberg’s false discovery rate were modified to balance the discovery of statistically significant genes with false-positive limitations. Probe sets without corresponding gene symbols were removed, and genes with multiple probes were averaged. The absolute values of logFC (fold change) > 0.1 and* P* < 0.1 were considered statistically significant. When we obtained data using the GEO database, we obtained the value of the gene logFC. The upregulated and downregulated genes were determined by the positive and negative values of the logFC values. Use the method above to get the upregulated and downregulated genes.

### Kyoto Encyclopedia of Genes and Genomes (KEGG) and Gene Ontology (GO) enrichment analyses of DEGs

The Database for Annotation, Visualization, and Integrated Discovery (DAVID; http://david.ncifcrf.gov) (6.8)^12^ is an online biological information database that integrates biological data and analysis tools to provide a comprehensive set of annotation information for functional genes and proteins. It provides users with a method to extract biological information. Additionally, KEGG (http://www.kegg.jp/kegg/kegg1.html) is a database resource for comprehending advanced functions and biological systems of large-scale molecular data generated by high-throughput experimental techniques^11,13,14^. Furthermore, GO (http://geneontology.org/) is a widely used bio-informatics technology for gene annotation and biological process analysis^15^. DAVID online database was established for biological analysis to analyze the function of DEG^16^.* P* < 0.05 was considered statistically significant according to the research.

### Protein–protein interaction (PPI) network construction and module analysis

We used the Search Tool for the Retrieval of Interacting Genes/Proteins (STRING; http://string-db.org) (version 10.0)^17^, an online database, to predict the PPI networks. The STRING database contains information on more than 5000 species, 20 million proteins, and 3 billion interactions. These protein interactions include both direct physical interactions and co-expression correlations. Through the STRING database, known protein–protein relationships can be identified to better understand the complex regulatory networks in organisms. Based on the previous studies, the analysis of functional interactions between proteins can provide new insights into the pathogenesis or development of diseases^18^. In this study, the PPI network of DEGs was established using the STRING database, and the interaction of a comprehensive score > 0.4 was considered statistically significant. It is noteworthy that Cytoscape (https://cytoscape.org/) (version 3.4.0) is an open-source bioinformatics software for visualizing molecular interaction networks^16^. Cytoscape is used to integrate biomolecular interaction networks with high-throughput gene expression data and other molecular states. It is most effective when combined with large databases of protein–protein, protein-DNA, and genetic interactions. The Molecular Complex Detection (MCODE, version 1.4.2) application in Cytoscape clusters a specified network based on its topology to search for pockets of dense communication^18^. MCODE is based on the relationship between edges and nodes in a huge network to identify key sub-networks and genes to facilitate downstream analysis. In this study, the PPI network was represented by Cytoscape, and the most significant module of the PPI network was identified by MCODE^19^. Additionally, the selection criteria were as follows: MCODE scores > 5, degree cut-off = 2, node score cut-off = 0.2, maximum depth = 100, and k-score = 2. To adapt DAVID to perform KEGG and GO analysis on DEGs, enrich GO and KEGG to obtain the top 3 pathways, and then a bioinformatics website was used to draw enriched point bubble maps for the pathways.

### Selection and analysis of hub genes

Among the important modules identified by the MCODE tool in Cytoscape software (http://www.cbioportal.org)^20^ are the extracted hub genes. We performed bioprocess analysis and visualized hub genes using Biological Networks Gene Oncology (BiNGO) version 3.0.3 tool: A Cytoscape plugin^21^. The core genes of this module were analyzed by KEGG and GO using DAVID, and the top three pathways were obtained using GO's biological process (BP)/molecular function (MF)/cell component (CC) and KEGG enrichment. We used the bioinformatics website to generate a focused dot bubble diagram.

## Results

### Identification of DEGs in patients with MDD

**Figure 2:**
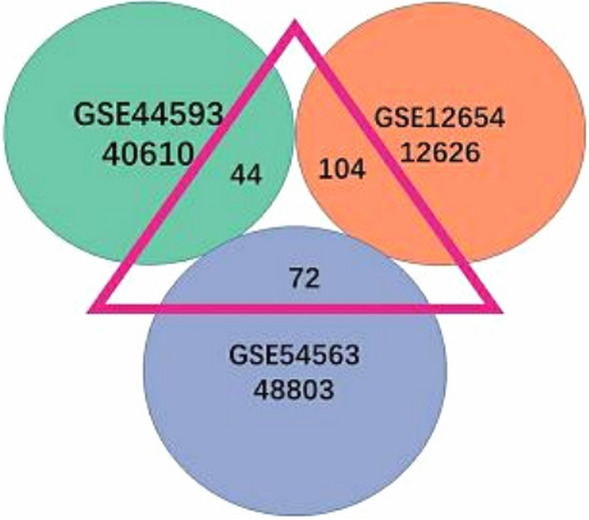
220 DEGs (44 genes were extracted from 40,610 genes in GSE445931, 104 genes were extracted from 12,626 genes in GSE12654, and 72 genes were extracted from 48,803 genes in GSE54563). The range of the number of genes within the pink triangle is: p < 0.05, logFC absolute value ≥ 0.1.

After normalizing the microarray results, 220 DEGs were identified (GSE445931: 44/40610; GSE12654: 104/12626; GSE54563: 72/48803). LogFC > − 0.1 and the absolute value of* P* < 0.1 are presented in the Wayne diagram (Fig. 2).

### PPI network construction and module analysis

**Figure 3:**
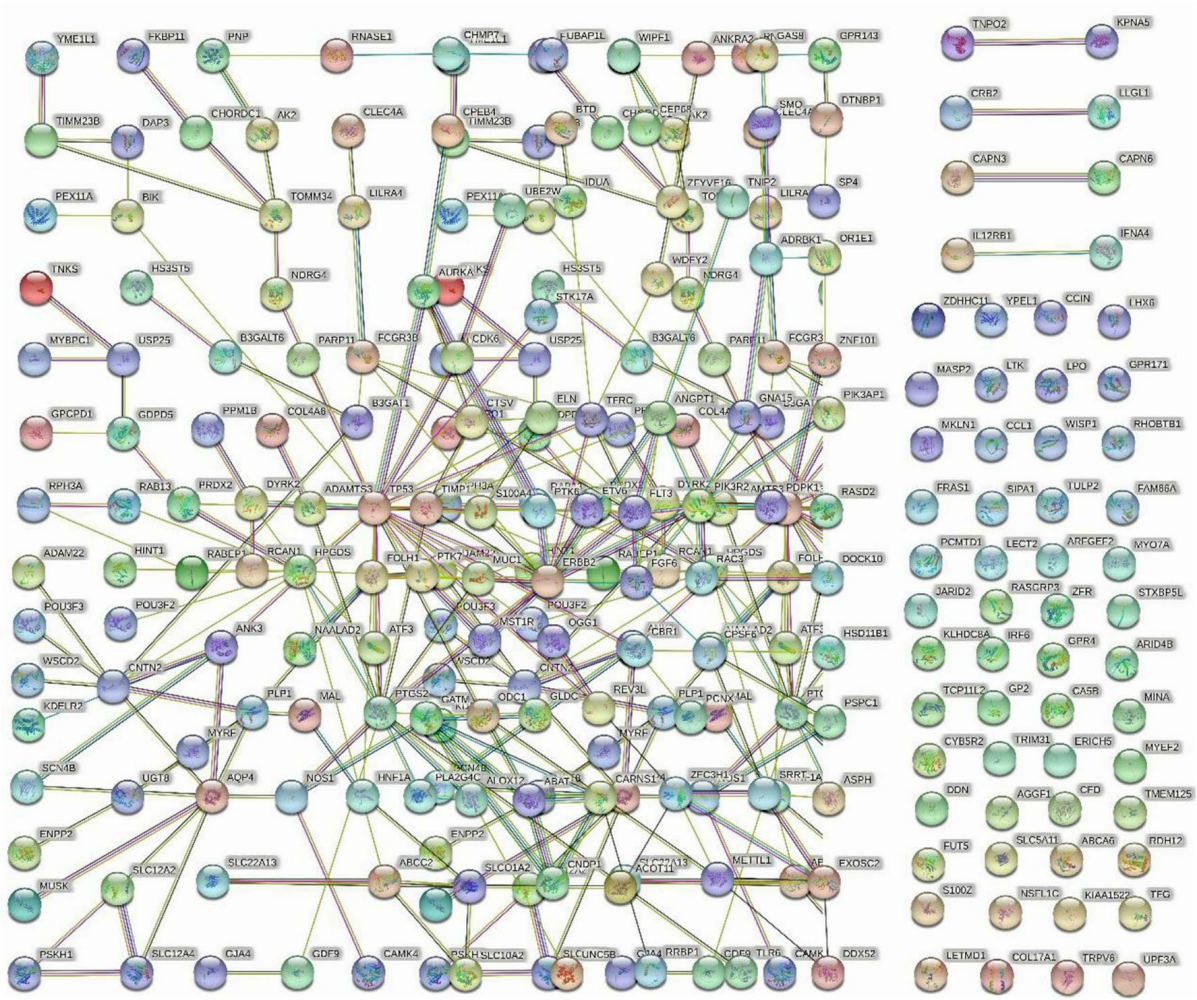
The protein-protien interaction (PPI) network of differentially expressed genes (DEGs) was constructed using STRING (link: http://string-db.org, version 10.0).

**Figure 4:**
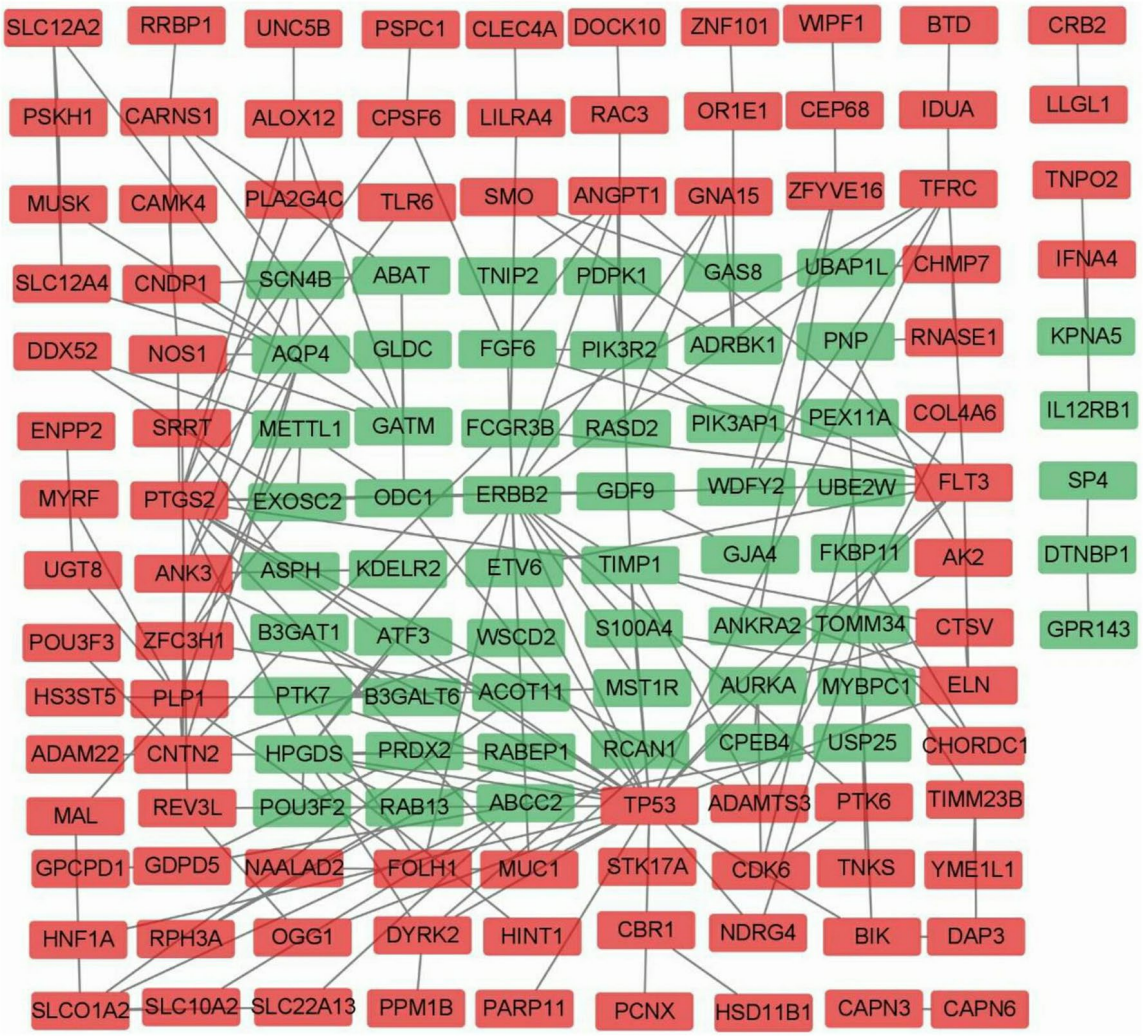
The protein-protien interaction (PPI) network of differentially expressed genes (DEGs) with upregulated and downregulated genes was constructed using Cytoscape (link: https://cytoscape.org/, version 3.4.0). Upregulated genes are marked in red; downregulated genes are marked in green.

**Figure 5:**
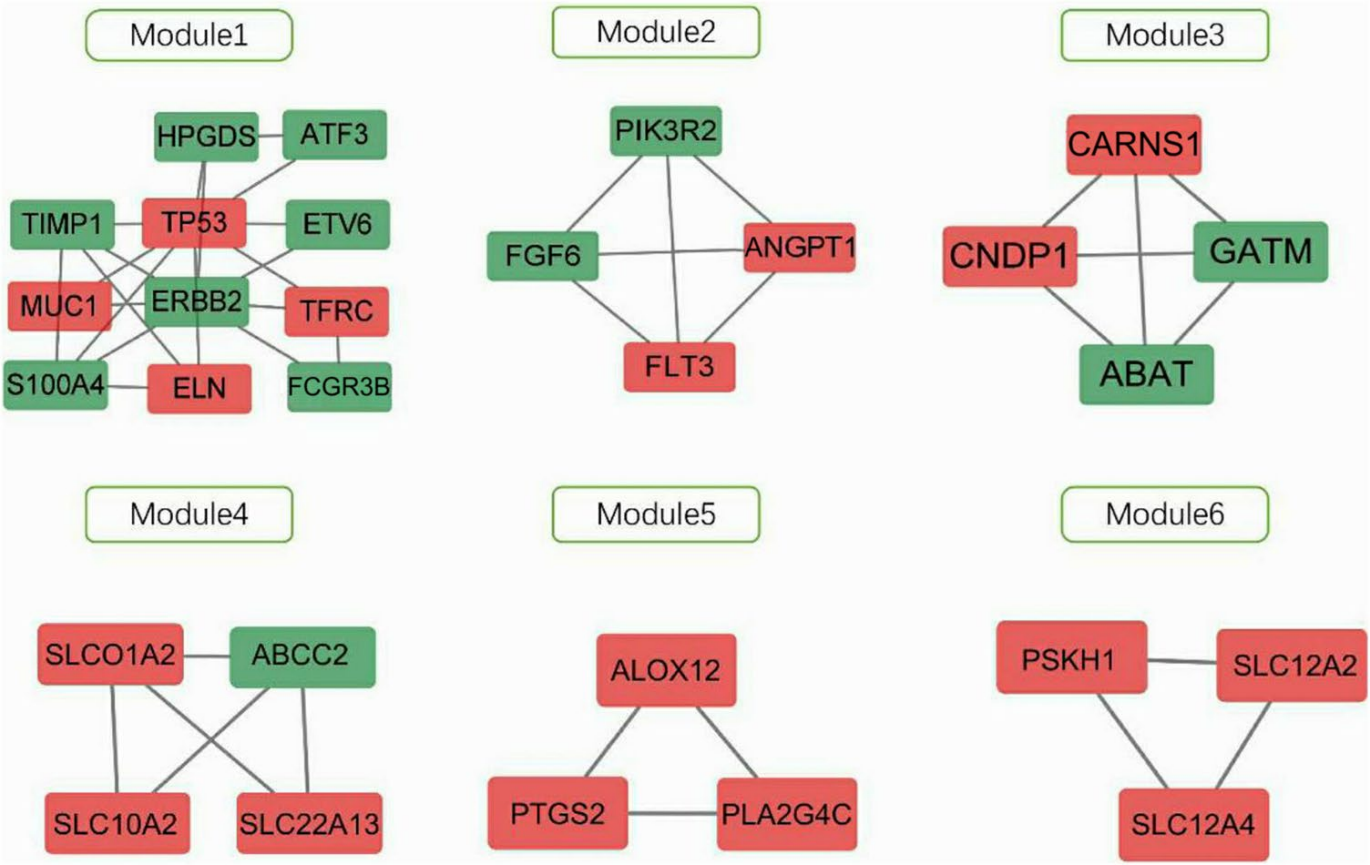
The six important modules of differentially expressed genes (DEGs).

The PPI network of DEGs consists of strings (https://string-db.org/) (Fig. 3), and the PPI network with DEGs with upregulated and downregulated genes was constructed using Cytoscape. Upregulated genes were indicated in red; downregulated genes were indicated in green (Fig. 4). Among them, the important modules were obtained by the MCODE software in Cytoscape version 1.4.2 (Fig. 5).

### GO and KEGG enrichment analysis of DEGs

**Figure 6:**
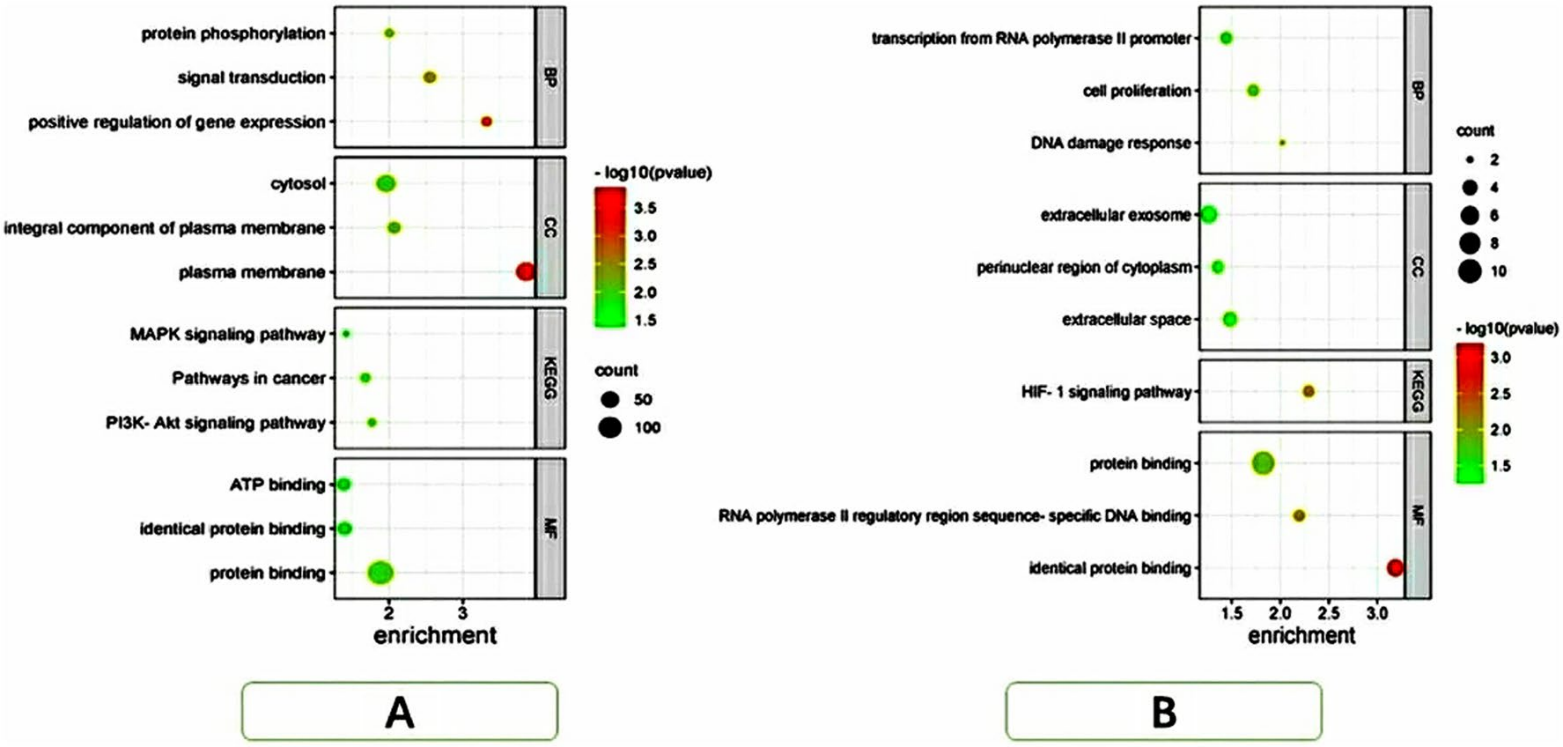
Gene Ontology (GO) enrichment and Kyoto Encylopedia of Genes and Genomes (KEGG) enrichment analyses were performed on differentially expressed genes (DEGs) and hub genes. GO enrichment of biological process (BP)/molecular function (MF)/cellular component (CC) and KEGG enrichment to obtain the top three pathways for drawing enrichment dot bubble. The size of the bubble indicates the enrichment score; colors indicate enrichment significane (KEGG, link: http://www.kegg.jp/kegg/keggl.html).

DAVID was used for function and pathway enrichment analysis to analyze the biological classification of DEG. GO analysis revealed that the changes in BP of DEGs were significantly enriched in the positive regulation of gene expression, signal transduction, and protein phosphorylation. The changes in MF of DEGs were mainly concentrated in the plasma membrane, components of the plasma membrane, and cytosol. The changes in CC of DEGs were prominently concentrated in protein binding, identical protein binding, and adenosine triphosphate (ATP) binding. According to the KEGG pathway analysis, DEGs were mainly enriched in three pathways, phosphatidylinositol-3-kinase-protein kinase B signaling pathway, cancer pathway, and mitogen-activated protein kinase signaling pathway (Table 1). The top three pathways were identified using an enrichment dot bubble diagram that was created using the bioinformatics website and GO-enriched BP/MF/CC and KEGG (Fig. 6A).

**Table 1:** GO and KEGG pathway enrichment analysis of DEGs in depression.

Category	Term	Count	%	P-value
GOTERM_BP_DIRECT	Signal transduction	23	11.5	2.80E−03
GOTERM_BP_DIRECT	Positive regulation of gene expression	15	7.5	4.80E−04
GOTERM_BP_DIRECT	Protein phosphorylation	12	6	9.90E−03
GOTERM_CC_DIRECT	Plasma membrane	70	35	1.40E−04
GOTERM_CC_DIRECT	Cytosol	66	33	1.10E−02
GOTERM_CC_DIRECT	Integral component of plasma membrane	24	12	8.50E−03
GOTERM_MF_DIRECT	Protein binding	143	71.5	1.30E−02
GOTERM_MF_DIRECT	Identical protein binding	26	13	4.00E−02
GOTERM_MF_DIRECT	ATP binding	24	12	4.10E−02
KEGG_PATHWAY	Pathways in cancer	14	7	2.10E−02
KEGG_PATHWAY	PI3K-Akt signaling pathway	11	5.5	1.70E−02
KEGG_PATHWAY	MAPK signaling pathway	9	4.5	3.80E−02

### Hub gene selection and analysis

**Figure 7:**
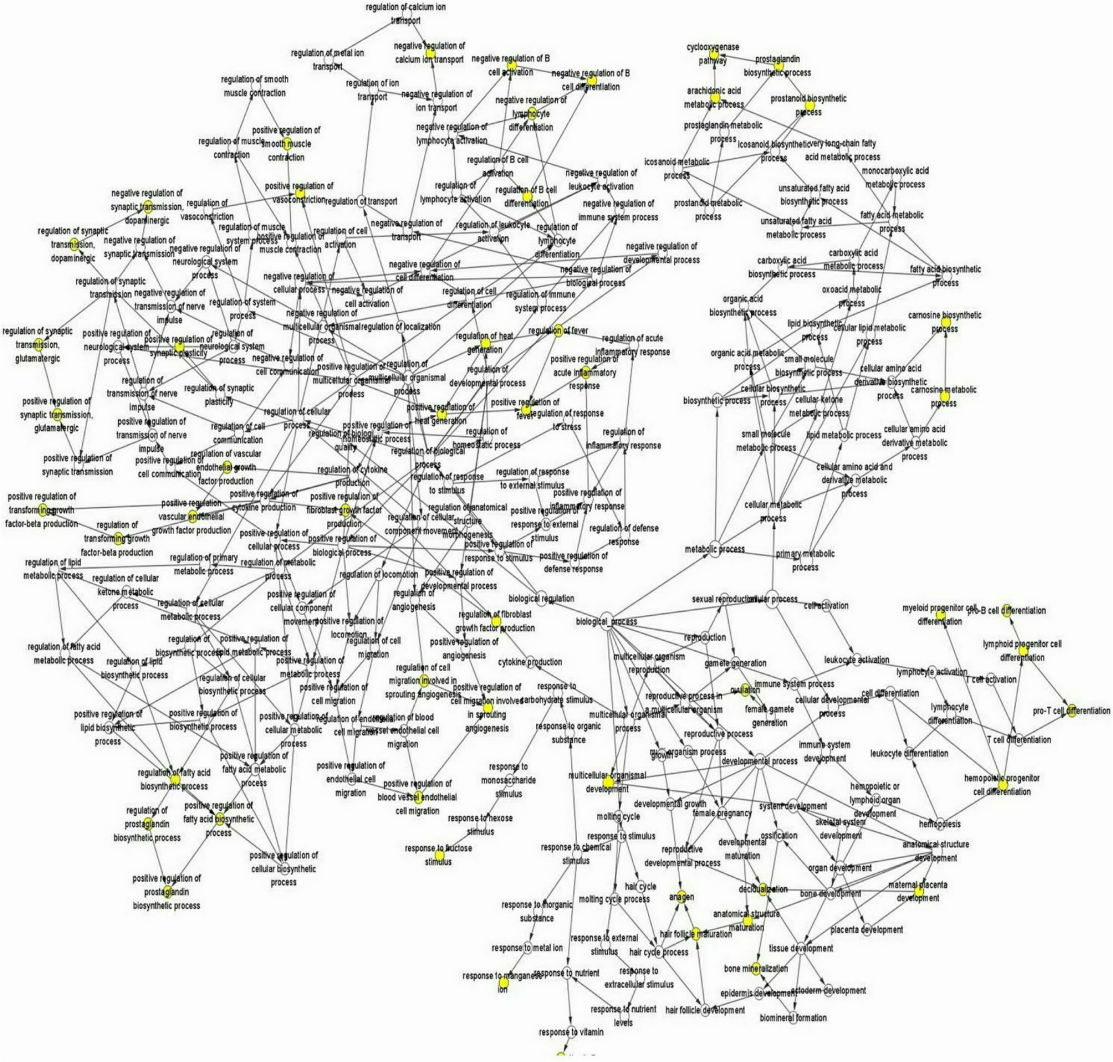
The biological process (BP) analysis and visualization of hub genes were performed using the Cytoscape BiNGO (link: https://cytoscape.org/, version 3.4.0).

Using an online platform, cBioPortal gene networks and co-expressed genes were analyzed (http://www.cbioportal.org)^18^. In the selection and analysis of Hub genes, a total of six genes were identified. The BP analysis and visualization of hub genes were done using the Cytoscape BiNGO (Fig. 7)^19^. See the supplementary material for detailed BP analysis node information. (Supplementary Information)

### KEGG and GO enrichment analysis of hub genes

Functional analysis of hub genes involved in important modules was analyzed using DAVID. The results revealed that the changes in gene BPs in this module were mainly concentrated in DNA damage response, signal transduction by the p53 class mediator causing transcription of p21 class mediator, cell proliferation, and transcription from RNA polymerase II promoter. Changes in MF were mainly concentrated in extracellular space, cytoplasmic perinuclear area, and extracellular exosomes. The changes in CC were mainly concentrated in the identical protein binding, RNA polymerase II regulatory region sequence-specific DNA binding, and protein binding. KEGG pathway analysis revealed the hypoxia-inducible factor 1 signaling pathway. GO and KEGG pathway enrichment analysis of DEG in the most significant module (Table 2). The top 3 pathways were identified using an enrichment dot bubble diagram that was created on the bioinformatics website and GO-enriched BP/MF/CC and KEGG (Fig. 6B).

**Table 2:** GO and KEGG pathway enrichment analysis of DEGs in the most significant module.

Category	Term	Count	%	P-value
GOTERM_BP_DIRECT	Positive regulation of transcription from RNA polymerase II promoter in response to endoplasmic reticulum stress	2	18.2	0.0071
GOTERM_BP_DIRECT	DNA damage response, signal transduction by p53 class mediator resulting in transcription of p21 class mediator	2	18.2	0.0095
GOTERM_BP_DIRECT	Cell proliferation	3	27.3	0.019
GOTERM_BP_DIRECT	Transcription from RNA polymerase II promoter	3	27.3	0.036
GOTERM_CC_DIRECT	Extracellular space	4	36.4	0.033
GOTERM_CC_DIRECT	Perinuclear region of cytoplasm	3	27.3	0.044
GOTERM_CC_DIRECT	Extracellular exosome	5	45.5	0.055
GOTERM_MF_DIRECT	Identical protein binding	5	45.5	6.50E−04
GOTERM_MF_DIRECT	RNA polymerase II regulatory region sequence-specific DNA binding	3	27.3	6.40E−03
GOTERM_MF_DIRECT	Protein binding	10	90.9	1.50E−02
KEGG_PATHWAY	HIF-1 signaling pathway	3	27.3	5.10E−03

## Discussion

Severe depression has recently been identified as a major threat to public health; millions of people suffer from this mental illness and die each year, and people aged 20–30 years are more likely to suffer from severe depression^22–24^. Notably, the number of female patients exceeds the number of male patients secondary to the influence of the interaction of physical, psychological, and social factors^25^. We used bioinformatic technology to identify the key genes of depression. Big data analysis allows for the selection of the depression genes that present the greatest promise for experimental confirmation, which not only speeds up and lowers the cost of biological experiments but also lowers overall research expenditure. Our main selection was the GEO dataset of human brain tissue, which also adds credibility.

In the case of MDD, a combination of antidepressants, antipsychotics, and psychotherapy or electroconvulsive therapy may be required. However, a lack of understanding about MDD or unwillingness to receive formal treatment among patients may have an adverse effect on the recovery rate in clinical treatment. Most patients are unable to gain a comprehensive understanding of MDD owing to its complexity^24^. There are many causes of depression, one of which is gene abnormality. Gene abnormalities include gene mutations such as protein genes, receptor genes, cytokine genes, and amino acid genes. A genomic report comparing the genomes of 135,000 people with depression with those of nearly 350,000 people without depression identified 44 independently significant loci associated with clinical features in patients with major depressive disorder^26^.

Additionally, since most hospitals are unable to provide personalized care, each patient must receive a unique course of treatment based on their unique neurobiological makeup. Therefore, we analyzed significant MDD genes based on the bioinformatic approaches to identify effective biomarkers and practical therapeutic targets. Potential markers are necessary for effective diagnosis and treatment. We can use microarray technology, which is considered a useful approach to discovering new biomarkers for other diseases, to identify the genetic changes in MDD^22^.

Meanwhile, three mRNA microarray datasets were used to compare the DEGs of people with and without MDD, and 220 DEGs were divided into different datasets, with 77 downregulated and 143 upregulated genes present in each dataset. We used GO and KEGG enrichment analysis to further study the interaction between DEGs.

We analyzed the important modules identified by the MCODE tool. The main genes present in Model 1 are TP53 ERBB2, MUC1, and ETV6, which are highly expressed in tumors, especially breast cancer^27^. Model 2 mainly contains PIK3R 2, FGF6, FLT3, and ANGPT1, which are mainly enriched in acute myeloid leukemia^28^. Model 3 primarily includes CARNS1, CNDP1, GATM, and ABAT, which are mainly distributed in tumors, particularly pancreatic cancer and breast cancer^29^. Model 4 mainly contains SLCO1A2, ABCC2, and SLC10A2, which are enriched in Alzheimer's disease and bile acid metabolism^30^. Module 5 mainly contains ALOX12, PTGS2, and PLA2G4C, which are enriched in the metabolism of endothelin and arachidonic acid^31^. Module 6 mainly contains PSKH1, SLC12A2, and SLC12A4, which are enriched in the development of the brain, particularly in the cerebellar cortex, hippocampus, and dorsomedial nucleus of the thalamus^32^.

We selected six hub genes. Among these central genes, the degrees of the node of electron transport system (ETS) variant transcription factor 6 (ETV6), FMS-related receptor tyrosine kinase 3 (FLT3), carnosine synthetase 1 (CARNS1), solute carrier family 22 member 13 (SLC22A13), prostaglandin endoperoxide synthetase 2 (PTGS2), protein serine kinase H1 (PSKH1), and ETV6 are high. The ETV6 gene encodes an ETS family transcription factor. Notably, ETV6 genes contain two functional domains: a DNA-binding domain at the C-terminus and an N-terminal pointed domain that contains PPI as well as other proteins^33^. Studies using gene knockouts have demonstrated that ETV6 plays a significant role in chromosomal rearrangements related to leukemia and congenital fibrosarcoma, influencing hematopoiesis, and maintaining the development of vascular networks^34,35^. Additionally, ETV6 is also closely linked to primary central nervous system lymphoma (PCNSL). More than 72 cases of PCNSL with normal immune function and a new diagnosis have been reported by Aurélie Bruno and Karim Labreche^36,37^.

FLT3 gene encodes type III receptor tyrosine kinase that regulates hematopoietic function. This receptor is initiated by the connection between the FMS-related tyrosine kinase 3 ligands and the extracellular domain^38^. According to an analysis by Adrien Tassou, Maxime Thouaye, and other academics and researchers, peripheral incisions may cause sensory and emotional changes via central pain sensitization. These modifications are associated with excessive microglia activation in the spinal cord, which can induce hypersensitivity in mice. The components of our model for neurotic speculation were simultaneously revealed by the increased expression of genes involved in axon regeneration in combination with neurotic markers. Studies have supported that peripheral FLT3, as a vital upstream neuromodulator of spinal microglial proliferation, is involved in central pain sensitization, which will cause excessive pain-related behaviors and depressive and anxiety disorders. Inhibition of FLT3 can become a promising treatment for pain sensitivity and related emotional changes^39^.

CARNS1 (EC 6.3.2.11) belongs to the ATP-grasp family which promotes the formation of myosin (β-alanine-l-histidine) and high myosin (γ-aminobutyric-l-histidine), primarily found in skeletal muscle and the central nervous system^40^. Li Zhang and Yan Zhang indicated that CARNS1 is remarkably downregulated in breast cancer. CARNS1 expression is closely related to tumor molecular and histological type T and M stages and survival status. Based on the Kaplan–Meier survival analysis, the downregulation of CARNS1 was significantly correlated with poor overall survival and relapse-free survival. Univariate and multivariate analysis demonstrated that CARNS1 can be considered an individual prognostic factor for patients with breast cancer. Additionally, experimental data suggested that CARNS1 protein and CARNS1 mRNA levels were significantly downregulated in breast cancer. Research has revealed that CARNS1, which is a tumor suppressor gene, can be considered an individual prognostic factor for patients with breast cancer^39^. Lívia de Souza Gonçalves and Lucas Peixoto Sales demonstrated that CARNS1 significantly impaired systolic function in the myocardium, using isolated and in vivo sarcomeres^40,41^. Systolic and diastolic dysfunction is strongly correlated with reduced intracellular Ca^2+^ peak and Ca^2+^ removal, but not with elevated oxidative stress markers or impaired mitochondrial respiration^42^. No increase in the amount of muscle myopeptide was observed following muscle peptide supplementation^43^.

SLC22A13, a member of the organic cation transporter family, codes for a transmembrane protein that transports small molecules and mediates the uptake of urate, a high-affinity niacin exchanger in the kidney and intestine^44^. Lactic acid is a nutritional substance frequently used as an intermediate in the synthesis of pyrimidines and is recommended for renal reabsorption. Uric acid transporter 1 (SLC22A12) is involved in tubular uptake^42^. Toshihide Higashino and Keito Morimoto et al. revealed that the dysfunctional variant of SLC22A13 reduced the risk of gout and serum uric acid levels, indicating that SLC22A13 is involved in the physiological reabsorption of uric acid salt in the human kidney^45^.

Prostaglandin-endoperoxide synthetase (PTGS), also called cyclooxygenase, is an important enzyme in prostaglandin biosynthesis and can be used as both dioxygenase and peroxidase. Additionally, PTGS has two isozymes: Constitutive PTGS1 and inducible PTGS2, which have different expression and tissue distribution control mechanisms. It is mainly responsible for the biosynthesis of prostaglandins and is involved in inflammation and mitosis^46,47^. In Basic and Clinical Medicine, it has been reported that the levels of PGE1 in plasma and brain tissue were measured by high-performance liquid chromatography and the mRNA levels of PLA2, PTGS1, PTGS2, and prostaglandin E synthetase were measured by quantitative reverse transcription polymerase chain reaction. Their findings revealed that PGE1 concentration and PTGS1 mRNA levels were significantly higher in the plasma of patients with MDD, and this result was verified in the blood and amygdala of the chronic unpredictable mild stress model of mice^48^.

PSKH1 has been suggested to enable protein kinase activity and play a crucial role in cardiac development and protein phosphorylation^49^. In this study, we defined that the expression of MicroRNA (miR)-566 was less in several colorectal cancer (CRC) cell lines (SW480, SW620, LoVo, HT29, and Caco-2) than that in the control colonic epithelial cell line (FHC). Data indicated that miR-566 can significantly inhibit the growth and metastasis of CRC cells by targeting PSKH1^50^.

## Conclusions

The interactions between MDD and the hub genes, ETV6, FLT3, CARNS1, SLC22A13, PTGS2, and PSKH1, have not been widely reported. ETV6 plays a key role in hematological diseases, breast cancer, and salivary gland cancer^26,37,51,52^. FLT3 inhibitor (tyrosine kinase inhibitor) and B-cell lymphoma-2 inhibitor coordinate and eliminate FLT3/ITD mutations of acute leukemia cells via B-cell lymphoma 2-like protein 11 activations^53^. The failure of TKI targeting FLT3-ITD in patients with AML to prevent a recurrence, despite complete remission, suggests resistance and/or persistence of leukemia initiation cells in the hematopoietic niche. It was demonstrated in vitro that FLT3-ITD AML cells have a decreased proliferative capacity when vacuolar protein sorting 34 is inhibited by simulating the hematopoietic ecology under low oxygen concentration^54,55^. CARNS1 mediates interferon gamma-induced arginine depletion in bovine mammary epithelial cells^40^. Miao and Ma demonstrated that CARNS1 may have a close relationship with muscle development^45^. Furthermore, CARNS1, which can also be an intermediate product of gout, may act as a candidate biomarker for the diagnosis and prognosis of clear cell renal cell carcinoma. Besides, PTGS2/nuclear factor kappa-light-chain-enhancer of activated B cell signaling pathway is involved in the radiation tolerance of glioma cells^56,57^. PSKH1 mediates the migration and invasion of CRC cells^58^. We also organized the key genes into hierarchical groups^59^. These core genes have been reported to distinguish samples from patients with MDD from those from patients without MDD, suggesting that they can be used as diagnostic biomarkers. These genes may play a key role in MDD development, progression, and recurrence.

MicroRNA (miRNA) is a class of non-coding RNA with a size of approximately 22 nt, which plays a key role in various biological processes related to human diseases^60^. Recent studies have indicated that miRNA is one of the epigenetic mechanisms that mediate the long-term effects of depression. MiRNAs have been proposed as biomarkers of depression that can identify high-risk patients with depression and predict their response to drug treatment^61^. Xing Chen et al. developed the computational model of Matrix Decomposition and Heterogeneous Graph Inference for miRNA-disease association prediction in 2018, which can discover new miRNA-disease associations by integrating prediction association probability, miRNA functional similarity, and disease semantic similarity profiles^62^. miRNAs are closely related to various human complex diseases, such as cancer and AIDS. Particularly, miRNAs can act as oncogenes or tumor suppressors in the occurrence and development of various cancer types, including breast cancer and prostate cancer. You, Zhu-Hong, et al. developed the Path-Based MiRNA-Disease Association (PBMDA) model. Using case studies, they discovered that PBMDA can be used as a powerful computational tool to accelerate the identification of disease-miRNA associations^63^. The computational model established by Xing Chen in 2019 has become an important means of identifying new miRNA-disease associations. The most promising miRNA-disease pairs can be selected for experimental validation, which reduce the time and cost of biological experiments. We can also put later in the diagnosis of depression^64^ that LingShe and FuxingLiu calculated viral similarity and drug similarity based on the genome sequence, chemical structure, and Gaussian association diagrams. Molecular docking and molecular dynamics simulations were performed to measure and screen anti-COVID-19 drugsyy^65^.

Conclusively, this study screened the genes of depression through the GEO dataset and demonstrated 220 DEGs and 6 hub genes, which provided a new perspective for the diagnosis and treatment of depression. The six central genes screened in this study may act as early diagnostic markers and important targets for depression and provide new targets and research directions for its diagnosis and treatment. The results of this study provide a theoretical basis for follow-up experimental research on depression. Only three datasets were selected in this study, and thus, more datasets must be included in the future. As scientific research advances, more GEO data will become available for researchers to analyze. With the advancement in deep learning technology in bioinformatics and artificial intelligence, we will further use better methods to analyze depression genes in the future. In this study, we mainly used bioinformatic technology for analysis and did not verify the identified genes, for which further experiments are needed (Supplementary Information).

## Supplementary Information

Supplementary Information.

## Data Availability

We would like to thank the staff of the GEO database for providing open-source and freely available data. The datasets generated and/or analyzed during this study are available in the GEO repository at https://www.ncbi.nlm.nih.gov/geo/.

## Abbreviations

BP: Biological process
CC: Cellular component
DEGs: Differentially expressed genes
GO: Gene Ontology
GEO: Gene Expression Omnibus
KEGG: Kyoto Encyclopedia of Genes and Genomes
MDD: Major depressive disorder
MF: Molecular function
NCBI: National Center for Biotechnology Information
PPI: Protein–protein interaction

## References

[1] Mccarron R, Shapiro B, Rawles J (2021). Depression. Ann. Intern. Med..

[2] Moussavi S (2007). Depression, chronic diseases, and decrements in health: Results from the World Health Surveys. Lancet.

[3] Herzog DP (2021). Early onset of depression and treatment outcome in patients with major depressive disorder. J. Psychiatr. Res..

[4] Zhang FF (2018). Brain structure alterations in depression: Psychoradiological evidence. CNS Neurosci. Ther..

[5] LeMoult J, Gotlib IH (2019). Depression: A cognitive perspective. Clin. Psychol. Rev..

[6] Ritchie ME (2015). limma powers differential expression analyses for RNA-sequencing and microarray studies. Nucleic Acids Res..

[7] Ron E, Michael D, Lash AE (2002). Gene Expression Omnibus: NCBI gene expression and hybridization array data repository. Nucleic Acids Res..

[8] Sibille E (2013). Molecular aging of the brain, neuroplasticity, and vulnerability to depression and other brain-related disorders. Dialogues Clin. Neurosci..

[9] Iwamoto K, Kakiuchi C, Bundo M, Ikeda K, Kato T (2004). Molecular characterization of bipolar disorder by comparing gene expression profiles of postmortem brains of major mental disorders. Mol. Psychiatry.

[10] Chang LC (2014). A conserved BDNF, glutamate-and GABA-enriched gene module related to human depression identified by coexpression meta-analysis and DNA variant genome-wide association studies. PLoS ONE.

[11] Xu Z (2016). Identification of candidate biomarkers and analysis of prognostic values in ovarian cancer by integrated bioinformatics analysis. Med. Oncol..

[12] Sherman BT (2007). The DAVID Gene Functional Classification Tool: A novel biological module-centric algorithm to functionally analyze large gene lists. Genome Biol..

[13] Kanehisa M (2019). Toward understanding the origin and evolution of cellular organisms. Protein Sci..

[14] Minoru K (2020). KEGG: Integrating viruses and cellular organisms. Nucleic Acids Res..

[15] Kanehisa, M. The KEGG database. (2002). 12539951

[16] Ashburner M (2000). Geneontology: Tool for the unification of biology. Nat. Genet..

[17] Franceschini A (2012). STRING v9.1: Protein–protein interaction networks, with increased coverage and integration. Nucleic Acids Res..

[18] Smoot ME, Ono K, Ruscheinski J, Wang P-L, Ideker T (2011). Cytoscape 2.8: New features for data integration and network visualization. Bioinformatics.

[19] Bandettini WP (2012). MultiContrast Delayed Enhancement (MCODE) improves detection of subendocardial myocardial infarction by late gadolinium enhancement cardiovascular magnetic resonance: A clinical validation study. J. Cardiovasc. Magn. Reson..

[20] Gao J (2013). Integrative analysis of complex cancer genomics and clinical profiles using the cBioPortal. Sci. Signal..

[21] Maere S, Heymans K, Kuiper M (2005). BiNGO: A cytoscape plugin to assess overrepresentation of gene ontology categories in biological networks. Bioinformatics.

[22] Jääskeläinen E (2018). Epidemiology of psychotic depression–systematic review and meta-analysis. Psychol. Med..

[23] Hollon SD (2019). Treatment of depression versus treatment of PTSD. Am. J. Psychiatry.

[24] Waitzfelder B (2018). Treatment initiation for new episodes of depression in primary care settings. J. Gen. Intern. Med..

[25] Shumaker SA, Hill DR (1991). Gender differences in social support and physical health. Health Psychol..

[26] Howard DM (2020). Major depressive disorder working group of the psychiatric genomics consortium. Nat. Commun..

[27] Bertucci F (2003). Breast cancer revisited using DNA array-based gene expression profiling. Int. J. Cancer.

[28] Hornick N (2013). Hypoxia regulates exosomal microrna content, trafficking and function of key elements in the AML microenvironment. Blood.

[29] Shen L (2022). Integrated application of transcriptome and metabolomics reveals potential therapeutic targets for the polarization of atherosclerotic macrophages. Biochim. Biophys. Acta Mol. Basis Dis.

[30] Ontsouka E (2021). Placental expression of bile acid transporters in intrahepatic cholestasis of pregnancy. Int. J. Mol. Sci..

[31] Bickford S (2014). Endothelin-1-mediated vasoconstriction alters cerebral gene expression in iron homeostasis and eicosanoid metabolism. Brain Res..

[32] Goran S (2016). Developmental expression patterns of KCC2 and functionally associated molecules in the human brain. Cereb. Cortex.

[33] Bohlander SK (2005). ETV6: A versatile player in leukemogenesis. Semin. Cancer Biol..

[34] Bruno A (2018). Identification of novel recurrent ETV6-IgH fusions in primary central nervous system lymphoma. Neuro Oncol..

[35] Park JC, Ashok A, Liu C, Kang H (2022). Real-world experience of NTRK fusion-positive thyroid cancer. JCO Precis. Oncol..

[36] Loo SK (2022). Fusion-associated carcinomas of the breast: Diagnostic, prognostic, and therapeutic significance. Genes Chromosomes Cancer.

[37] Fishman H (2022). ETV6-NCOA2 fusion induces T/myeloid mixed-phenotype leukemia through transformation of nonthymic hematopoietic progenitor cells. Blood J Am. Soc. Hematol..

[38] Gilliland DG, Griffin JD (2002). The roles of FLT3 in hematopoiesis and leukemia. Blood J. Am. Soc. Hematol..

[39] Tassou A (2021). Activation of peripheral neuronal FLT3 promotes exaggerated sensorial and emotional pain-related behaviors facilitating the transition from acute to chronic pain. bioRxiv.

[40] Zhang, M. *Mechanism of CARNS1-Mediated IFN-γ-Induced Arginine Depletion in Dairy Mammary Epithelial Cells* (Jilin University, 2020).

[41] Daver N, Schlenk RF, Russell NH, Levis MJ (2019). Targeting FLT3 mutations in AML: Review of current knowledge and evidence. Leukemia.

[42] Zhang L (2021). Combining bioinformatics analysis and experiments to explore CARNS1 as a prognostic biomarker for breast cancer. Mol. Genet. Genomic Med..

[43] de Souza Gonçalves L (2021). Histidine dipeptides are key regulators of excitation-contraction coupling in cardiac muscle: Evidence from a novel CARNS1 knockout rat model. Redox Biol..

[44] Bahn A (2008). Identification of a new urate and high affinity nicotinate transporter, hOAT10 (SLC22A13). J. Biol. Chem..

[45] Miao W (2021). Integrative ATAC-seq and RNA-seq analysis of the longissimus muscle of luchuan and duroc pigs. Front. Nutr..

[46] Higashino T (2020). Dysfunctional missense variant of OAT10/SLC22A13 decreases gout risk and serum uric acid levels. Ann. Rheum. Dis..

[47] Kosaka T (1994). Characterization of the human gene (PTGS2) encoding prostaglandin-endoperoxide synthase 2. Eur. J. Biochem..

[48] Ma MJ, Xu Q (2019). Increased prostaglandin E1 level in major depressive disorder is associated with depression-like behaviors. Basic Clin. Med..

[49] Brede G, Solheim J, Prydz H (2002). PSKH1, a novel splice factor compartment-associated serine kinase. Nucleic Acids Res..

[50] Zhang Y, Zhang S, Yin J, Xu R (2019). MiR-566 mediates cell migration and invasion in colon cancer cells by direct targeting of PSKH1. Cancer Cell Int..

[51] Skalova A (2018). Molecular profiling of mammary analog secretory carcinoma revealed a subset of tumors harboring a novel ETV6-RET translocation. Am. J. Surg. Pathol..

[52] Roberts KG (2018). ETV6-NTRK3 induces aggressive acute lymphoblastic leukemia highly sensitive to selective TRK inhibition. Blood J. Am. Soc. Hematol..

[53] Zhu R (2021). FLT3 tyrosine kinase inhibitors synergize with BCL-2 inhibition to eliminate FLT3/ITD acute leukemia cells through BIM activation. Signal Transduct. Target. Ther..

[54] Dupont M (2022). Autophagy targeting and hematological mobilization in FLT3-ITD acute myeloid leukemia decrease repopulating capacity and relapse by inducing apoptosis of committed leukemic cells. Cancers.

[55] Tien FM (2022). Distinct clinico-biological features in AML patients with low allelic ratio FLT3-ITD: Role of allogeneic stem cell transplantation in first remission. Bone Marrow Transplant..

[56] Kang W (2020). The SLC family are candidate diagnostic and prognostic biomarkers in clear cell renal cell carcinoma. Biomed. Res. Int..

[57] Suzuki E (2015). Transcriptional upregulation of HNF-1β by NF-κB in ovarian clear cell carcinoma modulates susceptibility to apoptosis through alteration in bcl-2 expression. Lab. Invest..

[58] Tan C (2019). Activation of PTGS2/NF-κB signaling pathway enhances radiation resistance of glioma. Cancer Med..

[59] Whitworth H (2012). Identification of kinases regulating prostate cancer cell growth using an RNAi phenotypic screen. PLoS ONE.

[60] Zheng X, Zhang C, Wan C (2022). MiRNA-Disease association prediction via non-negative matrix factorization based matrix completion. Signal Process..

[61] Louviere J, Timmermans H (2010). A review of recent advances in decompositional preference and choice models. Tijdschr. Econ. Soc. Geogr..

[62] Chen X, Yin J, Qu J (2018). MDHGI: Matrix decomposition and heterogeneous graph inference for miRNA-disease association prediction. PLoS Comput. Biol..

[63] You Z-H (2017). PBMDA: A novel and effective path-based computational model for miRNA-disease association prediction. PLoS Comput. Biol..

[64] Chen X (2019). MicroRNAs and complex diseases: From experimental results to computational models. Brief. Bioinform..

[65] Ling SA (2021). VDA-RWLRLS: An anti-SARS-CoV-2 drug prioritizing framework combining an unbalanced bi-random walk and Laplacian regularized least squares. Comput. Biol. Med..

